# Cervical spinal cord enlargement and systemic inflammation in diabetic peripheral neuropathy: a cross-sectional MRI study

**DOI:** 10.1007/s11845-026-04355-6

**Published:** 2026-04-08

**Authors:** Yaşar Taştemur, Şeyma Taştemur, İrfan Atik, Hacer Baş Ekici, Deniz Apalan, Vedat Sabancioğullari

**Affiliations:** 1 Department of Anatomy, Faculty of Medicine, Sivas Cumhuriyet University, 58140 Sivas, Türkiye; 2 Department of Internal Medicine, Faculty of Medicine, Sivas Cumhuriyet University, 58140 Sivas, Türkiye; 3 Department of Radiology, Faculty of Medicine, Sivas Cumhuriyet University, 58140 Sivas, Türkiye; 4 Department of Anatomy, Faculty of Veterinary Medicine, Sivas Cumhuriyet University, 58140 Sivas, Türkiye

**Keywords:** Diabetic peripheral neuropathy, Magnetic resonance imaging, Neuroinflammation, Spinal cord, Systemic immune-inflammation index, Type 2 diabetes mellitus

## Abstract

**Background:**

Diabetic peripheral neuropathy (DPN) is one of the most common and disabling complications of type 2 diabetes mellitus (T2DM). Although traditionally considered a peripheral disorder, recent evidence indicates concomitant central nervous system (CNS) involvement. The systemic immune-inflammation index (SII) has emerged as a biomarker reflecting inflammatory activity in diabetes. This study aimed to assess cervical spinal cord cross-sectional area (CSA) using magnetic resonance imaging (MRI) in diabetic patients with and without DPN and to examine its association with systemic inflammation and metabolic parameters.

**Methods:**

This retrospective, cross-sectional study included 299 participants: 100 healthy controls, 100 diabetic patients without DPN, and 99 with DPN. Cervical spinal CSA was measured on MRI, and fasting plasma glucose (FPG), glycated hemoglobin (HbA1c), complete blood count parameters, and SII values were analyzed. Intergroup comparisons and correlations were evaluated using nonparametric tests and multivariate models.

**Results:**

Both diabetic groups showed significantly higher HbA1c, FPG, and SII levels compared with the control group (*p* < 0.05). Cervical spinal CSA was significantly greater in diabetic groups, particularly in those with DPN (*p* = 0.004). CSA correlated weakly and positively with HbA1c and FPG, but not with SII.

**Conclusion:**

Diabetic patients, especially with DPN, exhibit cervical spinal cord enlargement accompanied by elevated systemic inflammation. The CSA increase may represent an early phase of neuroinflammation preceding atrophy. Combining MRI-derived CSA with SII may improve early detection and risk stratification, supporting an integrated assessment of central and peripheral neuroinvolvement in diabetes.

## Introduction

Diabetes mellitus (DM) is a chronic metabolic disorder with a rapidly increasing global prevalence, with type 2 diabetes mellitus (T2DM) representing the vast majority of cases [1]. Among its microvascular complications, diabetic peripheral neuropathy (DPN) remains a significant cause of morbidity and mortality. DPN typically manifests as a distal symmetric polyneuropathy, beginning in the extremities and presenting with symptoms such as burning, stabbing, or cramping pain, as well as paresthesia and allodynia [2, 3]. Once established, DPN is difficult to reverse and may lead to severe consequences such as foot ulceration, Charcot arthropathy, and amputation [4]. While traditionally regarded as a purely peripheral nerve disorder, accumulating evidence indicates that DPN may also involve the central nervous system (CNS), including the spinal cord. The initial discovery of spinal cord atrophy in DPN was initially interpreted as a secondary consequence of distal axonopathy, often described as a “dying-back” phenomenon. Early MRI investigations demonstrated a significant reduction in the cervical spinal cord cross-sectional area in patients with advanced DPN compared with non-diabetic controls [5]. In one of the earliest structural studies, spinal cord atrophy was quantified at the C2/C3 intervertebral disc level, establishing a reproducible reference point for morphometric assessment [6]. Subsequent work revealed that spinal cord involvement could also be detected in patients with subclinical DPN, suggesting that central injury may accompany—or even precede—peripheral nerve damage [7]. More recent research further supports the concept that the neurological impact of diabetes extends beyond peripheral nerves to include central nervous system structures. Notably, MRI evidence has demonstrated early spinal cord alterations in diabetic individuals even before overt neuropathic symptoms emerge [8]. Neuroimaging studies have reported cervical spinal cord atrophy and microstructural alterations in individuals with DPN compared with diabetic patients without neuropathy and healthy controls [9, 10].

Chronic low-grade inflammation has emerged as a key mechanism linking hyperglycemia to diabetic complications. The systemic inflammatory index (SII)—derived from platelet, neutrophil, and lymphocyte counts—has been associated with the onset of T2DM, metabolic control, and progression [11]. Elevated SII values have also been correlated with diabetic microvascular complications such as nephropathy and retinopathy, underscoring the central role of systemic inflammation in diabetes pathophysiology [12, 13].

Despite these insights, few studies have examined the potential relationship between spinal cord structural changes and systemic inflammatory indices in diabetic populations—particularly when distinguishing between those with and without DPN. Given that cervical spinal cord cross-sectional area (CSA) measured by MRI serves as a non-invasive marker of spinal involvement, and SII provides a simple yet powerful indicator of systemic inflammation, exploring their relationship is of clinical importance. Accordingly, this study aimed (1) to compare cervical spinal cord CSA among healthy controls, T2DM patients without DPN, and T2DM patients with DPN, and (2) to investigate associations between spinal cord CSA, metabolic control markers glycated hemoglobin (HbA1c), fasting plasma glucose), hematologic indices, and SII. We hypothesized that participants with DPN would show reduced spinal cord CSA and elevated SII compared to non-neuropathic and control groups, and that CSA would be inversely associated with systemic inflammation and poor glycemic control.

## Materials and methods

This retrospective, cross-sectional study was conducted in collaboration among the Departments of Internal Medicine, Anatomy, and Radiology. The study was approved by the Ethics Committee of Sivas Cumhuriyet University Health Sciences Research Board (Approval No: 2025–04/56; Date: April 24, 2025) and was conducted in accordance with the principles outlined in the Declaration of Helsinki. All participants’ personal data were kept strictly confidential.

A total of 299 individuals who underwent cervical spinal magnetic resonance imaging (MRI) at our institution between 2020 and 2025 and had complete laboratory data were included. Participants were divided into three groups: (i) Control group – individuals without a diagnosis of diabetes mellitus (*n* = 100), (ii) Diabetic group with DPN – patients diagnosed with T2DM and DPN (*n* = 99), and (iii) Diabetic group without DPN – patients diagnosed with T2DM but without DPN (*n* = 100).

The diagnosis of DPN was retrospectively verified from patient records based on clinical symptoms, neurological examination findings, and, where available, electrophysiological test results.

The inclusion criteria were an age of over 18 years and the availability of complete MRI and laboratory data within the study timeframe. Individuals with acute infection, systemic inflammatory disease, malignancy, hematologic disorders, multiple sclerosis (MS) or other destructive neurological disorders, spinal cord pathology, or poor-quality MRI images were excluded from the study.

**Fig. 1:**
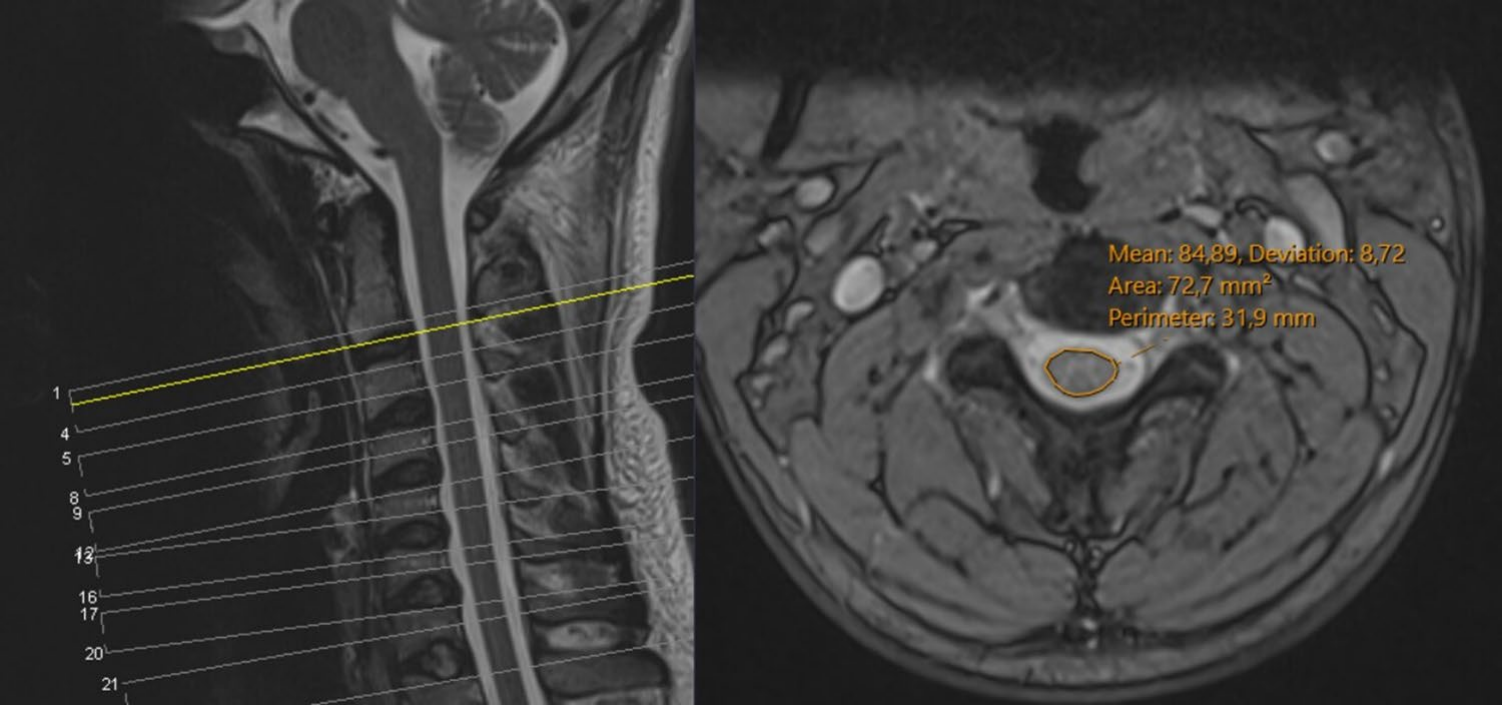
Measurement of the cervical spinal cord cross-sectional area at the C2–C3 level using axial T2-weighted MRI

Cervical MRI scans were retrieved retrospectively from the institutional Picture Archiving and Communication System (PACS). All images were obtained using 1.5 Tesla MRI scanners following standardized acquisition protocols. The CSA of the cervical spinal cord (mm^2^) was measured manually on axial T2-weighted images at the C2–C3 intervertebral disc level. Manual contouring was performed using a region-of-interest (ROI) technique that encompassed the entire spinal cord cross-section (Fig. 1). All measurements were independently performed by two experienced radiologists (with 9 and 5 years of neuroradiology experience, respectively) who were blinded to the clinical and laboratory data. In addition, spinal cord signal intensity was visually assessed on both T1- and T2-weighted images in cases with CSA differences. No abnormal signal intensity changes were identified in the evaluated spinal cord segments. Interobserver agreement was assessed using the intraclass correlation coefficient (ICC) to ensure measurement reliability.

Laboratory findings — HbA1c, fasting plasma glucose (FPG), neutrophil, lymphocyte, platelet, and hemoglobin levels — obtained within ± 1 month of the MRI date (i.e., within one month before or after imaging) were included in the analysis. The SII was calculated using the following formula:

$$\mathrm{SII}=\frac{\text{Neutrophil count} \left({10}^{3}/\mu \mathrm{L}\right)\times \text{Platelet count }\left({10}^{3}/\mu \mathrm{L}\right)}{\text{Lymphocyte count }\left({10}^{3}/\mu \mathrm{L}\right)}$$

### Statistical analysis

All statistical analyses were performed using IBM SPSS Statistics for Windows, Version 27.0 (IBM Corp., Armonk, NY, USA). The distribution of continuous variables was assessed using the Shapiro–Wilk test. Data following a normal distribution were analyzed with one-way analysis of variance (ANOVA), and pairwise comparisons were conducted using the Bonferroni post hoc test. For non-normally distributed variables, the Kruskal–Wallis test was applied, followed by Dunn’s post hoc test to identify specific group differences.

The effects of group (control, diabetic without DPN, diabetic with DPN) and gender on the dependent variables were evaluated using a two-way ANOVA. Both main effects and interaction effects (group × gender) were examined. The assumption of homogeneity of variances was verified using Levene’s test.

To address potential confounding, supplementary analyses were performed using analysis of covariance (ANCOVA) models adjusted for age and gender, with cervical CSA as the dependent variable and group as the fixed factor. Estimated marginal means with 95% confidence intervals were reported.

Associations between continuous variables were examined using Spearman’s rank correlation coefficient (ρ), which assesses monotonic relationships without requiring normality. To determine the independent predictors of the cervical spinal cord cross-sectional area, a multiple linear regression analysis was conducted.

All tests were two-tailed, and a *p*-value < 0.05 was considered statistically significant. Continuous variables are expressed as mean ± standard error (SE) and standard deviation (SD), as appropriate.

## Results

The demographic, biochemical, and hematological characteristics of the participants are summarized in Table 1. Significant differences were observed among the groups in terms of age, HbA1c, FPG, neutrophil count, systemic inflammatory index (SII), and cervical spinal cord cross-sectional area (*p* < 0.05). The control group exhibited significantly lower mean values of age, HbA1c, and FPG compared with both diabetic groups (*p* < 0.001). The cross-sectional area of the cervical spinal cord was higher in the diabetic groups than in the controls, with the highest mean value observed in the diabetic group with peripheral neuropathy (*p* = 0.004). When adjusted for age and gender, the group effect on CSA remained significant, although the effect size was small to moderate (partial η^2^ ≈ 0.03). This pattern indicates that the observed differences are not solely attributable to differences in age or gender. Similarly, neutrophil count and SII were significantly elevated in both diabetic groups relative to the control group (*p* < 0.001 and *p* = 0.004, respectively). No significant differences were detected among the groups in lymphocyte count, hemoglobin, or platelet count (*p* > 0.05). For variables showing substantial differences, post-hoc pairwise comparisons were performed using Dunn’s test (Table 1).

**Table 1:** Comparison of demographic and clinical parameters among the study groups

Variable	Control Group (*n* = 100)	DPN Group (*n* = 99)	non-DPN Group (*n* = 100)	*p*-value
Age (years)	52.15 ± 0.98ᵃ (SD: 9.70)	61.15 ± 0.77ᵇ (SD: 7.55)	58.78 ± 0.97ᵇ (SD: 9.54)	< 0.001*
Cervical spinal cord cross-sectional area (mm^2^)	74.36 ± 1.04ᵃ (SD: 10.34)	78.18 ± 1.12ᵃᵇ (SD: 11.10)	79.30 ± 1.08ᵇ (SD: 10.80)	0.004*
HbA1c (%)	5.74 ± 0.04ᵃ (SD: 0.39)	8.19 ± 0.14ᵇ (SD: 1.35)	7.84 ± 0.12ᵇ (SD: 1.15)	< 0.001*
Fasting plasma glucose (mg/dL)	98.84 ± 1.42ᵃ (SD: 14.10)	188.21 ± 6.60ᵇ (SD: 65.72)	160.32 ± 4.73ᵇ (SD: 46.13)	< 0.001*
Neutrophil count (10^3^/μL)	4.02 ± 0.12ᵃ (SD: 1.11)	5.01 ± 0.14ᵇ (SD: 1.40)	4.64 ± 0.13ᵇ (SD: 1.30)	< 0.001*
Lymphocyte count (10^3^/μL)	2.40 ± 0.06 (SD: 0.64)	2.29 ± 0.06 (SD: 0.61)	2.36 ± 0.07 (SD: 0.68)	0.580
Hemoglobin (g/dL)	14.03 ± 0.16 (SD: 1.60)	14.10 ± 0.13 (SD: 1.33)	14.23 ± 0.17 (SD: 1.68)	0.584
Platelet count (10^3^/μL)	267.20 ± 6.43 (SD: 64.02)	267.33 ± 6.94 (SD: 67.27)	276.42 ± 6.50 (SD: 64.73)	0.485
Systemic Inflammatory Index (SII)	485.09 ± 22.09ᵃ (SD: 214.16)	585.73 ± 24.87ᵇ (SD: 243.74)	536.33 ± 21.30ᵃᵇ (SD: 206.48)	0.004*

*p*-values were calculated using the Kruskal–Wallis test. “ *” indicates a statistically significant difference (*p* < 0.05). Superscript letters (a, b, c) denote pairwise group comparisons identified by the Dunn post hoc test (*p* < 0.05)

The two-way analysis of variance (ANOVA) for cervical spinal cord cross-sectional area revealed a significant main effect of gender (F₁,₂₉₂ = 15.97, *p* < 0.001, *η*^2^ = 0.052). The detailed results are summarized in Table 2. The group factor showed an approaching significance (F₂,₂₉₂ = 3.01, *p* = 0.051), while the group × gender interaction was not statistically significant (*p* = 0.629). Bonferroni post-hoc tests demonstrated that the control group differed significantly from both diabetic groups, whereas no significant difference was observed between the DPN group and the non-DPN group. According to Levene’s test, the assumption of homogeneity of variances was met (*p* = 0.053), confirming the validity of the ANOVA results.

**Table 2:** Two-way ANOVA results for cervical spinal cord cross-sectional area (descriptive statistics and post-hoc comparisons)

Source/Comparison	df	F	*p*	Partial η^2^	Mean ± SD
Group	2	3.010	0.051	0.020	Control group: 74.37 ± 10.34 DPN Group: 78.19 ± 11.13 non-DPN Group: 79.30 ± 10.80
Gender	1	15.965	< 0.001	0.052	Female (*n* = 181): 75.06 ± 9.95 Male (*n* = 118): 80.74 ± 11.52
Group × Gender	2	0.465	0.629	0.003	—
Post-hoc (Bonferroni)	—	—	—	—	Control group – DPN group = –3.82 (*p* = 0.033) Control group – non-DPN group = –4.93 (*p* = 0.003) DPN group – non-DPN group = –1.11 (*p* = 1.000)

Dependent variable: *Cervical spinal cord cross-sectional area. p* < 0.05 was considered statistically significant. (df = degrees of freedom; F = F-statistic; p = probability value; η^2^ = partial eta-squared (effect size); SD = standard deviation; DPN = Diabetic peripheral neuropathy)

The two-way ANOVA results for HbA1c and FPG are presented in Table 3. A significant main effect of group was found for both parameters (HbA1c: F₂,₂₈₂ = 153.18, *p* < 0.001, *η*^2^ = 0.521; FPG: F₂,₂₈₇ = 103.07, *p* < 0.001, *η*^2^ = 0.418). The main effect of gender was not significant for HbA1c (*p* = 0.223), whereas a weak but significant effect was observed for FPG (*p* = 0.028). For both variables, the group × gender interaction was significant (HbA1c: *p* = 0.001; FPG: *p* < 0.001), indicating that gender-related differences varied across groups. Bonferroni post-hoc tests revealed that the control group had significantly lower HbA1c and FPG values compared with both diabetic groups. In contrast, intergroup differences between the DPN group and the non-DPN group were not significant for HbA1c but significant for FPG (*p* < 0.001).

**Table 3:** Two-way ANOVA and post-hoc test results for HbA1c and fasting plasma glucose levels

Parameter	Source/Comparison	df	F	*p*	Partial η^2^	Mean ± SD
HbA1c (%)	**Group**	2	153.184	< 0.001	0.521	Control group: 5.74 ± 0.39 DPN group: 8.19 ± 1.35 non-DPN group: 7.84 ± 1.15
	**Gender**	1	1.494	0.223	0.005	Female: 7.09 ± 1.51 Male: 7.46 ± 1.48
	**Group × Gender**	2	7.081	0.001	0.048	—
	**Post-hoc (Bonferroni)**	—	—	—	—	Control group–DPN group: –2.45 (*p* < 0.001) Control group–non-DPN group: –2.11 (*p* < 0.001) DPN group–non-DPN group: + 0.35 (*p* = 0.058)
FPG (mg/dL)	**Group**	2	103.070	< 0.001	0.418	Control group: 98.85 ± 14.10 DPN group: 188.21 ± 65.73 non-DPN group: 160.33 ± 46.13
	**Gender**	1	4.870	0.028	0.017	Female: 145.36 ± 54.04 Male: 157.59 ± 67.40
	**Group × Gender**	2	10.910	< 0.001	0.071	—
	**Post-hoc (Bonferroni)**	—	—	—	—	Control group–DPN group: –89.36 (*p* < 0.001) Control group–non-DPN group: –61.48 (*p* < 0.001) DPN group–non-DPN group: + 27.89 (*p* < 0.001)

*Fasting plasma glucose (FPG). p* < 0.05 was considered statistically significant. Homogeneity of variances was confirmed by Levene’s test (*p* = 0.053). (df = degrees of freedom; F = F-statistic; η^2^ = partial eta-squared (effect size); SD = standard deviation; DPN = Diabetic peripheral neuropathy)

A significant main effect of group was observed in the two-way ANOVA analysis for neutrophil counts (F₂,₂₈₂ = 8.11, *p* < 0.001, *η*^2^ = 0.054), indicating that neutrophil levels differed significantly across study groups (Table 4). The group × gender interaction for neutrophils was also significant (F₂,₂₈₂ = 4.06, *p* = 0.018, *η*^2^ = 0.028), demonstrating that the effect of gender on neutrophil levels varied by group. The main effect of gender was not significant (*p* = 0.262). For lymphocyte counts, no significant main or interaction effects were detected (*p* > 0.05), suggesting similar distributions across groups and genders. In contrast, platelet counts showed a significant main effect of gender (F₁,₂₈₇ = 11.37, *p* = 0.001, *η*^2^ = 0.038), with higher mean platelet levels in females; however, neither the group effect (*p* = 0.198) nor the interaction (*p* = 0.878) reached significance. Regarding SII, the group effect approached significance (F₂,₂₈₅ = 2.99, *p* = 0.052, *η*^2^ = 0.021), while the main effect of gender (*p* = 0.316) and the interaction (*p* = 0.345) were not significant.

**Table 4:** Descriptive statistics and two-way anova results for neutrophil, lymphocyte, platelet, and systemic inflammatory index levels

Parameter	Group	Female (Mean ± SD)	Male (Mean ± SD)	Total (Mean ± SD)	Source of Variance	df	F	*p*	Partial η^2^
Neutrophil (× 10⁹/L)	**Control group**	3.42 ± 0.74	3.89 ± 0.68	3.66 ± 0.72	Group	2	8.11	< 0.001	0.054
	**DPN group**	3.78 ± 0.69	4.02 ± 0.73	3.90 ± 0.71	Gender	1	1.27	0.262	0.004
	**non-DPN group**	4.00 ± 0.71	4.45 ± 0.80	4.21 ± 0.76	Group × Gender	2	4.06	0.018	0.028
Lymphocyte (× 10⁹/L)	**Control group**	2.15 ± 0.58	2.42 ± 0.65	2.28 ± 0.61	Group	2	1.31	0.271	0.009
	**DPN group**	2.29 ± 0.62	2.53 ± 0.67	2.41 ± 0.65	Gender	1	3.48	0.063	0.012
	**non-DPN group**	2.32 ± 0.60	2.57 ± 0.64	2.45 ± 0.62	Group × Gender	2	1.66	0.192	0.011
Platelet (× 10^3^/µL)	**Control group**	270 ± 54	305 ± 61	287 ± 58	Group	2	1.63	0.198	0.011
	**DPN group**	281 ± 59	317 ± 63	299 ± 61	Gender	1	11.37	0.001	0.038
	**non-DPN group**	289 ± 55	322 ± 58	306 ± 57	Group × Gender	2	0.13	0.878	0.001
SII	**Control group**	72.77 ± 10.33	78.22 ± 9.48	74.37 ± 10.34	Group	2	2.99	0.052	0.021
	**DPN group**	76.98 ± 9.55	80.48 ± 13.52	78.19 ± 11.13	Gender	1	1.01	0.316	0.004
	**non-DPN group**	75.83 ± 9.43	82.25 ± 11.10	79.30 ± 10.80	Group × Gender	2	1.07	0.345	0.008

Dependent variables: *neutrophil, lymphocyte, platelet, and systemic inflammatory index (SII) levels. p* < 0.05 was considered statistically significant. Homogeneity of variances was assessed using Levene’s test; the assumption was met in all analyses except for neutrophils, where minor deviation did not affect robustness due to balanced sample sizes. (df = degrees of freedom; F = F-statistic; η^2^ = partial eta-squared (effect size); SD = standard deviation; DPN = Diabetic peripheral neuropathy; SII = Systemic Inflammatory Index)

Correlation analyses between cervical spinal cord CSA and selected clinical and biochemical parameters are presented in Table 5. Spearman’s rank correlation analysis revealed a weak but statistically significant positive correlation between cervical spinal cord CSA and hemoglobin levels (*ρ* = 0.197, *p* < 0.001), as well as weaker positive correlations with HbA1c (*ρ* = 0.137, *p* = 0.020) and fasting plasma glucose (*ρ* = 0.117, *p* = 0.046). No significant correlation was observed between CSA and age. Cervical spinal cord CSA was not significantly correlated with neutrophil, lymphocyte or platelet counts or with the SII (*p* > 0.05). Among all examined variables, the strongest correlation was observed between HbA1c and FPG (*ρ* = 0.855, *p* < 0.001), consistent with expected glycemic associations. Moderate correlations were also observed between neutrophil count and SII, and between lymphocyte count and SII, indicating internal consistency among inflammatory markers.

**Table 5:** Spearman’s correlation analysis between cervical spinal cord cross-sectional area and clinical/biochemical parameters

Variable Pair	ρ (Rho)	*p*-value	Strength & direction
Age – Lymphocyte	–0.169	0.004*	Weak negative
Age – Hemoglobin	–0.139	0.018*	Weak negative
Age – Platelet	–0.129	0.029*	Weak negative
Area – HbA1c	0.137	0.020*	Weak positive
Area – FPG	0.117	0.046*	Weak positive
Area – Hemoglobin	0.197	< 0.001**	Weak positive
HbA1c – FPG	0.855	< 0.001**	Very strong positive
HbA1c – Neutrophil	0.240	< 0.001**	Weak positive
FPG – Neutrophil	0.251	< 0.001**	Weak positive
FPG – SII	0.128	0.033*	Weak positive
Neutrophil – SII	0.661	< 0.001**	Moderate positive
Neutrophil – Platelet	0.200	< 0.001**	Weak positive
Lymphocyte – Platelet	0.339	< 0.001**	Weak positive
Lymphocyte – SII	–0.183	0.002*	Weak negative
Hemoglobin – Platelet	–0.194	< 0.001**	Weak negative
Area – SII	0.030	0.616	Not significant
Area – Neutrophil	0.099	0.092	Not significant
Area – Lymphocyte	0.110	0.061	Not significant (approaching significance)

Spearman’s rho (ρ) correlation coefficients are shown. Age (years), HbA1c (%), fasting plasma glucose (FPG, mg/dL), hemoglobin (g/dL), platelet (× 10^3^/µL), lymphocyte and neutrophil counts (× 10^3^/µL), systemic inflammatory index (SII, arbitrary units), and cervical spinal cord cross-sectional area (mm^2^). Asterisks indicate significance levels (*p* < 0.05; *p* < 0.01). Asterisks indicate significance levels (*p* < 0.05; *p* < 0.01). ρ = Spearman’s correlation coefficient; (+) = positive correlation; (–) = negative correlation. Weak (|ρ|< 0.3), moderate (0.3 ≤|ρ|< 0.6), strong (|ρ|≥ 0.6). (HbA1c = glycated hemoglobin; FPG = fasting plasma glucose; SII = systemic inflammatory index; ρ = Spearman’s rank correlation coefficient)

Overall, these findings suggest that age and gender, as well as inflammatory parameters, do not strongly drive variation in cervical spinal cord CSA; instead, the observed differences appear to be more closely associated with group-level, disease-related factors.

## Discussion

DPN is one of the most prevalent and debilitating complications of diabetes, contributing significantly to long-term disability and reduced quality of life [11]. The pathogenesis of DPN is multifactorial, involving hyperglycemia-induced oxidative stress, mitochondrial dysfunction, and advanced glycation end-product (AGE)-mediated inflammatory cascades [14]. Increasing evidence challenges the notion that DPN is limited to peripheral nerve injury, suggesting that CNS structures, particularly the spinal cord, may also be affected. MRI studies have demonstrated cervical spinal cord CSA reduction and microstructural abnormalities in patients with DPN, supporting a model of combined peripheral and central neurodegeneration [10, 15]. Diffusion tensor imaging further reinforces this concept, revealing white matter changes correlated with neuropathic severity [16].

Contrary to several previous MRI-based studies reporting spinal cord atrophy in DPN patients, the present study demonstrated a modest but significant increase in cervical spinal CSA among both diabetic and DPN groups compared with healthy controls. Earlier investigations, such as those by Zang et al. (2023) and Stam et al. (2019), observed reduced CSA and microstructural degeneration suggestive of central axonal loss and demyelination in DPN [9, 17]. However, other recent work has proposed that spinal cord enlargement may occur in earlier or metabolically active stages of diabetes, potentially reflecting inflammatory edema, astrocytic hypertrophy, or glial activation preceding neurodegenerative shrinkage. This interpretation aligns with the hypothesis that diabetes-related neuroinflammation could transiently increase tissue volume before chronic ischemic and demyelinating processes induce atrophy [18, 19]. Hyperglycemia-induced microvascular dysfunction may lead to increased capillary permeability and vasogenic edema within spinal tissue, while metabolic overload and mitochondrial stress could trigger astrocytic hypertrophy and glial activation. These vascular and glial mechanisms are consistent with recent evidence highlighting endothelial dysfunction as a key driver of diabetic microangiopathy and tissue edema in central neural structures [20].

Furthermore, the observed CSA enlargement may represent early neuroinflammatory alterations rather than structural degeneration. Experimental and clinical evidence support that chronic hyperglycemia can trigger microglial activation, astrocytic hypertrophy, and interstitial edema within spinal tissue, leading to transient volumetric expansion before axonal degeneration [21, 22]. Notably, the relationship between neuroinflammation and glycemic dysfunction appears bidirectional—neuroinflammatory activation may not only result from hyperglycemia. However, it can also exacerbate insulin resistance and metabolic dysregulation through cytokine-mediated hypothalamic and autonomic pathways [23, 24]. Collectively, these findings support a potential “early inflammatory expansion–late atrophy” model of diabetic spinal cord involvement, wherein neuroinflammation, glial activation, and microvascular ischemia jointly shape the trajectory of structural alterations.

In our study, the significant group differences observed in HbA1c and FPG levels align with emerging evidence linking the metabolic burden of diabetes to neuroaxial structural changes. Recent longitudinal cohort and neuroimaging investigations have demonstrated that elevated HbA1c levels are associated with progressive alterations in brain structure—particularly increases in white matter hyperintensity volume (WMH) and cortical thinning—even within prediabetic ranges of glycemia. Specifically, Shin and colleagues analyzed a sample of 30,579 adults and identified a negative association between HbA1c and cerebral cortical thickness within the prediabetic range. In a separate study, Schweitzer et al. found that higher HbA₁c levels were significantly associated with greater two-year progression of WMH in a South Korean cohort [25, 26].

Chronic hyperglycaemia cumulatively promotes cerebral small‐vessel disease and neurodegeneration through mechanisms such as oxidative stress, mitochondrial dysfunction, and chronic inflammation, resulting in accelerated CNS structural and functional decline [27]. Although the association between spinal cord CSA and HbA1c was weakly positive in our dataset, this finding can be interpreted within a pathophysiological framework in which worsening glycemic control precipitates an early neuroinflammatory/edematous expansion phase—followed later by neurodegeneration and atrophy. FPG, which reflects more short- to mid-term glycaemic exposure, may also relate to broader CNS glucose metabolism disruption and changes in network connectivity, as reported by recent functional and imaging studies [28]. In sum, our findings suggest that group differences in HbA1c and FPG are consistent with the concept of a glycaemic paralysis of CNS homeostasis, where glucose dysregulation elicits microvascular and neuroinflammatory changes that progressively impact structural integrity. The weak but present correlation between CSA and HbA1c supports the view that CSA alteration may represent an early structural marker of this dynamic pathological cascade. Given that metabolic dysregulation and inflammation are closely intertwined in diabetes, it is also relevant to examine how systemic inflammatory burden, as reflected by the SII, relates to these morphometric alterations. In our study, SII levels demonstrated a clear gradient across groups, with the lowest levels in controls, intermediate levels in diabetic patients without DPN, and the highest levels among those with DPN. This pattern aligns with recent evidence suggesting that the systemic inflammatory burden increases in tandem with more advanced or complicated diabetes phenotypes [29]. For example, an extensive Chinese cross-sectional study found that individuals in the highest quartile of SII had a significantly increased prevalence of DPN compared with those in the lowest quartile [30].

A similar pattern is observed with microvascular complications. In diabetic retinopathy (DR) cohorts, SII is associated with both presence and severity, and some studies have reported that SII (and related indices) provides diagnostic/predictive performance for DR; this supports the higher SII pattern we observed in our DPN subgroup [30–33]. In diabetic kidney disease, higher SII has also been reported to be independently associated with disease presence and severity, and to have biomarker potential for distinguishing phenotypes; these findings reinforce our interpretation that the overall systemic inflammatory burden increases with more complicated diabetes phenotypes.

From a biological perspective, the underlying SII has become clearer in recent years, with the interaction between peripheral immune response and central neuroinflammation in DPN becoming more evident. The central role of microglial activation in DPN pathogenesis and its bidirectional feeding with peripheral inflammatory signals (e.g., neutrophilic activity, platelet-mediated mechanisms) may biologically explain the increase in peripheral blood-based indices (such as SII). Current review and mechanistic studies emphasize that DPN is shaped by the systemic interaction of metabolic, vascular, and inflammatory axes; this framework is consistent with our finding of higher SII in the DPN subgroup [29, 34–37].

The correlation trends observed in our study align with the emerging literature, which emphasizes the inflammatory component of diabetes. The moderate positive association between neutrophil count and SII is expected, given that neutrophils are a principal component of this index and reflect innate immune activation. Recent studies have confirmed that elevated neutrophil-derived indices, including SII, are linked to poor glycemic control and heightened risk of diabetic microvascular complications [38, 39].

The weak yet positive correlations between SII and HbA1c or FPG observed in our cohort suggest an early interplay between metabolic dysregulation and systemic inflammation. Consistent with this, population-based data indicate that SII tends to increase with worsening glycemic status and insulin resistance, even before overt complications appear [40]. Such findings support the notion that hyperglycemia-induced immune activation may precede measurable neurostructural alterations, linking metabolic burden to inflammatory signaling cascades in both peripheral and central tissues.

Taken together, the stepwise rise in SII across study groups supports its value as a biomarker reflecting systemic inflammatory amplification in diabetic neuropathic phenotypes. This pattern reinforces the evolving view that DPN is not merely a localized peripheral pathology but part of a broader systemic inflammatory state contributing to neurodegenerative progression.

The weak but significant correlations observed between cervical spinal cord CSA and metabolic–hematologic parameters (HbA1c, fasting plasma glucose, and hemoglobin) suggest early vascular and metabolic stress rather than established neurodegeneration. Chronic hyperglycemia may impair spinal microcirculation through endothelial dysfunction and reduced erythrocyte deformability, resulting in subtle hypoxia and metabolic strain within the cord tissue [41, 42].

Meanwhile, the weak association between CSA and inflammatory indices, such as the SII or neutrophil count, suggests that peripheral inflammation may not directly mirror central neuroinflammatory activity. Recent imaging evidence suggests that the blood–spinal cord barrier restricts peripheral immune translocation, resulting in a temporal dissociation between systemic and central inflammation [43, 44]. Collectively, these findings suggest that metabolic and microvascular factors may precede or dominate over overt inflammation in the early neuroaxial involvement of diabetes.

In our study, we observed that the cervical spinal cord CSA and platelet values were slightly higher in female participants compared to males. This difference is consistent with the gender-related physiological variations described in the literature. The regulatory effects of estrogen on endothelial function, microcirculation, and hematopoietic activity in women may provide a biological basis for these structural and hematological differences. Furthermore, estrogen’s antioxidant, neuroprotective, and neuroinflammation-suppressing properties may contribute to better preservation of spinal cord integrity in women, even under diabetic metabolic stress [45, 46].

Our findings suggest that the relative elevation in CSA observed in women may be related to neurovascular protective mechanisms. Similarly, recent neuroimaging studies have reported higher normalized CSA values and milder structural shrinkage in women within diabetic populations [47]. However, the magnitude of these differences is limited and requires support from advanced analyses that account for variables such as hormonal status, age, and diabetes duration.

In clinical practice, measurement of cervical spinal cord CSA may serve as a valuable adjunct biomarker. While conventional nerve conduction studies primarily evaluate peripheral nerve function, CSA quantification via MRI provides insight into central nervous system involvement in DPN. In this study, CSA effectively differentiated between controls, diabetic patients without DPN, and those with DPN, suggesting that it could help identify early spinal cord alterations even before overt neuropathic symptoms emerge. Spinal MRI, therefore, holds potential not only for early detection but also for risk stratification and prognosis [48]. For instance, individuals with preserved metabolic control but reduced CSA might warrant closer follow-up, whereas increasing CSA in the early inflammatory phase or a subsequent reduction in CSA in the degenerative phase could reflect disease progression. Integrating these measurements into longitudinal protocols could establish CSA as both a research metric and a surrogate clinical endpoint. Moreover, combining MRI-derived CSA with simple, cost-effective biomarkers such as SII may enhance diagnostic precision and practicality. Elevated SII values may prompt an MRI evaluation, while imaging results can inform targeted management strategies. This dual-marker approach—linking systemic inflammation with central morphometric changes—may support personalized risk profiling, earlier intervention, and improved outcomes in patients with diabetic peripheral neuropathy.

## Limitations

This study has several limitations that should be considered when interpreting the results. First, its retrospective and cross-sectional design limits the ability to infer causality. The diagnosis of DPN was based on retrospective clinical documentation, which may introduce a risk of misclassification. Although intra- and interobserver reliability for CSA measurements was verified using intraclass correlation coefficients (ICC), complementary agreement analyses—such as Bland–Altman plots—would provide additional insight into measurement variability. Furthermore, several potential confounders—including diabetes duration, body mass index, use of antihyperglycemic or anti-inflammatory medications, hypertension, and lipid profile—were not included in the multivariable adjustment models. These factors may partly account for individual differences in CSA or inflammatory indices. In addition, although differences in age and gender distribution existed between groups, correlation analyses demonstrated no significant association between cervical spinal cord CSA and age and only weak associations with metabolic parameters, suggesting that the observed CSA differences are unlikely to be driven solely by age, gender or systemic inflammatory status. Future prospective studies incorporating standardized DPN diagnostics, detailed metabolic and pharmacologic data, and longitudinal multimodal imaging are needed to validate and expand these findings. Taken together, while our results support the proposed “early inflammatory expansion–late atrophy” trajectory in diabetic spinal cord involvement, this mechanistic interpretation remains speculative. It requires confirmation through larger, longitudinal datasets and advanced imaging modalities.

## Conclusion

This study demonstrates that individuals with T2DM, particularly those with DPN, exhibit measurable alterations in cervical spinal cord morphology accompanied by increased systemic inflammatory activity. The mild yet significant enlargement in spinal cord CSA may represent an early neuroinflammatory or vasogenic phase preceding degenerative atrophy, aligning with evolving models of diabetes-related neurodegeneration that encompass both central and peripheral mechanisms. The modest associations observed between glycemic and inflammatory markers suggest that metabolic dysregulation and systemic inflammation jointly contribute to this neuroaxial involvement. Taken together, these findings support the hypothesis of an “early inflammatory expansion–late atrophy” trajectory in diabetic spinal cord pathology, which should be further validated through longitudinal and multimodal imaging studies integrating metabolic, inflammatory, and morphometric assessments.

## Data Availability

The datasets used and/or analyzed during the current study are available from the corresponding author on reasonable request.
